# Autographa californica Multiple Nucleopolyhedrovirus Ac34 Protein Retains Cellular Actin-Related Protein 2/3 Complex in the Nucleus by Subversion of CRM1-Dependent Nuclear Export

**DOI:** 10.1371/journal.ppat.1005994

**Published:** 2016-11-01

**Authors:** Jingfang Mu, Yongli Zhang, Yangyang Hu, Xue Hu, Yuan Zhou, He Zhao, Rongjuan Pei, Chunchen Wu, Jizheng Chen, Han Zhao, Kai Yang, Monique M. van Oers, Xinwen Chen, Yun Wang

**Affiliations:** 1 State Key Laboratory of Virology, Wuhan Institute of Virology, Chinese Academy of Sciences, Wuhan, China; 2 University of Chinese Academy of Sciences, Beijing, China; 3 State Key Laboratory of Biocontrol, Sun Yat-sen University, Guangzhou, China; 4 Laboratory of Virology, Wageningen University, Wageningen, the Netherlands; University of Guelph, CANADA

## Abstract

Actin, nucleation-promoting factors (NPFs), and the actin-related protein 2/3 complex (Arp2/3) are key elements of the cellular actin polymerization machinery. With nuclear actin polymerization implicated in ever-expanding biological processes and the discovery of the nuclear import mechanisms of actin and NPFs, determining Arp2/3 nucleo-cytoplasmic shuttling mechanism is important for understanding the function of nuclear actin. A unique feature of alphabaculovirus infection of insect cells is the robust nuclear accumulation of Arp2/3, which induces actin polymerization in the nucleus to assist in virus replication. We found that Ac34, a viral late gene product encoded by the alphabaculovirus Autographa californica multiple nucleopolyhedrovirus (AcMNPV), is involved in Arp2/3 nuclear accumulation during virus infection. Further assays revealed that the subcellular distribution of Arp2/3 under steady-state conditions is controlled by chromosomal maintenance 1 (CRM1)-dependent nuclear export. Upon AcMNPV infection, Ac34 inhibits CRM1 pathway and leads to Arp2/3 retention in the nucleus.

## Author Summary

Actin is one of the most abundant molecules in eukaryotic cells. Actin polymerization is a process that nucleates actin monomers into filamentous structures, and this cellular process is frequently used by viruses to facilitate virus multiplication in host cells. Arp2/3, the central regulator of actin polymerization, is predominantly localized in the cytoplasm under steady-state conditions. Alphabaculoviruses assemble their progeny nucleocapsids in the nucleus of host cells, and this process is heavily dependent on nuclear actin polymerization, which requires the virus to accumulate Arp2/3 in the nucleus. Yet, how baculovirus retains Arp2/3 in the nucleus remained largely unknown. In this study, we found that the distribution of Arp2/3 is dependent on CRM1, a receptor located on the nuclear membrane that mediates the export of a large number of proteins from the nucleus to the cytoplasm. AcMNPV protein Ac34 can inhibit the CRM1 function, and lead to Arp2/3 retention in the nucleus to assist in virus replication.

## Introduction

Actin polymerization is an evolutionarily conserved biological process in eukaryotic cells. The key elements of cellular actin polymerization machinery include, but are not limited to, actin, nucleation promoting factors (NPFs), and the actin-related protein 2/3 complex (Arp2/3). Arp2/3 was first isolated from *Acanthamoeba castellani* [1] and consists of seven subunits, including Arp2, Arp3, P40/ARPC1 (P40), P34/ARPC2 (P34), P21/ARPC3 (P21), P20/ARPC4 (P20), and P16/ARPC5 (P16) (Reviewed in [2, 3]). Activated by NPFs, Arp2/3 initiates globular actin (G-actin) polymerization into filamentous actin (F-actin) (Reviewed in [4]). Under steady-state conditions, Arp2/3 and other actin polymerization elements are predominantly localized in the cytoplasm. However, increasing evidence has shown that actin polymerization elements are also present in the nucleus and play important roles ranging from chromatin remodeling to transcription regulation (Reviewed in [5, 6]). The nuclear import mechanisms of actin and N-WASP, one of the best characterized NPFs, were previously determined [7–10], whereas nucleo-cytoplasmic shuttling mechanism of Arp2/3 remains enigmatic.

Intracellular pathogens, such as *Listeria monocytogenes* [11], *Rickettsia spp*. [12], vaccinia virus [13], alpha-herpesvirus [14], human immunodeficiency virus [15], and *Burkholderia thailandensis* [16], frequently use the host actin polymerization machinery to assist in pathogen reproduction (Reviewed in [17–20]). Alphabaculovirus is thus far the smallest pathogen known to profit from the host actin polymerization machinery for their propagation [21–23]. After the host cell entry of the Autographa californica multiple nucleopolyhedrovirus (AcMNPV), one of the best-characterized alphabaculoviruses, cellular Arp2/3 is activated by P78/83, a virus-encoded NPF [23]. In this way, P78/83 induces cytoplasmic actin polymerization to propel nucleocapsid migration towards the nucleus, where viral genome replication, gene transcription, and nucleocapsid assembly occur [21, 24]. However, unlike most pathogens that induce primarily cytoplasmic actin polymerization, AcMNPV also induces nuclear actin polymerization, which is essential for nucleocapsid assembly in the nucleus and for progeny nucleocapsid transport to the nuclear periphery [22, 23, 25–28]. The unique feature of nuclear actin polymerization induced by AcMNPV requires the accumulation of the cytoplasmic actin polymerization machinery, including Arp2/3, in the nucleus [27, 29–31], which makes this virus-infection system ideally suited as a research model for investigating the nucleo-cytoplasmic shuttling mechanism of Arp2/3.

Chromosomal maintenance 1 (CRM1), also known as exportin-1, is a highly versatile transport receptor in eukaryotic cells. In the nucleus, CRM1 binds to its cargo protein, usually harboring a nuclear export sequence (NES) containing a leucine-rich motif L_xxx_L_xx_L_x_L, along with RanGTP, to form a CRM1-cargo-RanGTP complex [32]. This complex interacts with several nucleoporins within the nuclear pore complex (NPC) and migrates across the NPC to the cytoplasm (Reviewed in [33]). After its nuclear export, RanGTP is hydrolyzed to RanGDP, and the complex releases the cargo protein to the cytosol.

In this research, we found that Arp2/3 subcellular distribution is controlled by CRM1-dependent nuclear export under steady-state conditions. AcMNPV infection induced Arp2/3 nuclear retention by inhibiting the CRM1 pathway with a viral late gene product, Ac34. To our knowledge, this is the first study describing the nuclear retention mechanism of Arp2/3 under steady-state and virus-infection conditions. We also provide the first example of a virus specifically blocking the CRM1 nuclear export pathway to promote its replication.

## Results

### An AcMNPV late gene product induces P40 nuclear accumulation

Previously, we and other groups have revealed the nuclear accumulation mechanism of P78/83 and G-actin [29–31], two key elements of the actin polymerization machinery, during AcMNPV infection. To investigate how AcMNPV accumulates Arp2/3, the central regulator of actin polymerization, in the nucleus, we cloned the cDNA sequences of Arp2/3 subunits from Sf9 cells, a commercially available *Spodoptera frugiperda* cell line commonly used for baculovirus infection (GenBank Accession: KJ187399.1, JQ364941.1, KJ187400.1, GU356595.1, KJ187401.1, KJ187402.1) [34]. Here, P40 was selected to represent Arp2/3 because P40 appeared to be the most abundant protein detected by either Western blot or fluorescence microscopy (Arp2 and P20 were less abundant than P40; Arp2 could only be detected by Western blot; other subunits were barely detected by Western blot or fluorescence microscopy when transiently expressed in Sf9 cells). We prepared plasmid-based expression constructs encoding P40 tagged with a V_5_ epitope (P40-V_5_) at its C-terminus or P40 fused to enhanced green fluorescent protein at its N-terminus (EGFP-P40) to monitor the Arp2/3 dynamics during AcMNPV infection.

**Fig 1:**
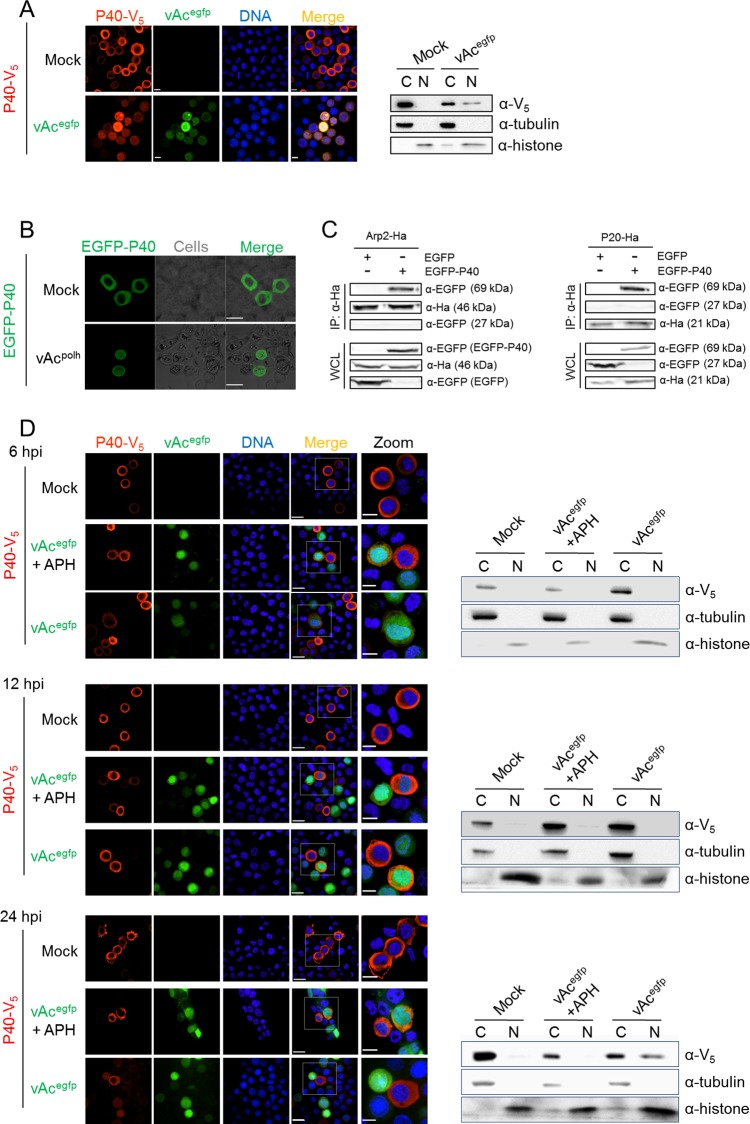
P40 subcellular distribution in AcMNPV-infected cells. (A). Subcellular distribution of P40 in plasmid-transfected cells. P40-V_5_ was transiently expressed in Sf9 cells by plasmid transfection and then the cells were either mock-infected or infected with vAc^egfp^ (MOI = 2). At 24 hpi, the cells were subjected to either immunofluorescence microscopy (left panel) or cell fractionation (right panel). For immunofluorescence microscopy, the cells were fixed, probed with anti-V_5_ antibody to detect P40-V_5_, and visualized with Alexa Fluor 568-conjugated secondary antibody (red). Hoechst 33258 was used to stain the nuclear DNA (blue). For the cell fractionation assay, the cells were harvested and separated into nuclear (N) and cytoplasmic (C) fractions. The fractions were separated by SDS-PAGE and probed with anti-V_5_, anti-tubulin, and anti-histone-H_3_ antibodies to detect P40, tubulin, and histone-H_3_, respectively. (B). EGFP-P40 subcellular distribution. EGFP-P40 was expressed in mock-infected Sf9 cells or cells infected with vAc^polh^ (MOI = 2), a bacmid derived from the AcMNPV genome with an inserted polyhedrin open reading frame (ORF). At 48 hpt, the cells were analyzed by fluorescence microscopy. (C). EGFP-P40 associates with Arp2 and P20. EGFP (approx. 27 kDa) or EGFP-P40 (approx. 69 kDa) was co-expressed with Arp2-Ha (approx. 46 kDa) or P20-Ha (approx. 21 kDa) in Sf9 cells, respectively. At 48 hpt, the cells were harvested and subjected to a co-IP assay using anti-Ha. The immunoprecipitated proteins and the WCL were separated by SDS-PAGE and probed with anti-Ha and anti-EGFP. (D). AcMNPV late gene products induce P40 nuclear accumulation in the late infection phase. Sf9 cells transfected with the P40-V_5_ encoding plasmid were either mock infected or infected with vAc^egfp^ (MOI = 2) in the presence or absence of APH. The cells were fixed at 6, 12 and 24 hpi or harvested for fractionation and further treated as described in (A). Scale bar: 20 μm; Scale bar in the zoomed panel: 10 μm.

Cytoplasmic localization was noted by immunofluorescence for P40-V_5_ for mock infected cells (Fig 1A, left panel), but some nuclear localization was observed for cells infected with AcMNPV carrying an EGFP marker (vAc^egfp^, diagramed in S1A Fig). As evidenced by cell fraction and Western blot, P40-V_5_ was present in only the cytoplasmic fraction of mock infected cells, while some P40-V_5_ was found in the nuclear fraction of vAc^egfp^ infected cells (Fig 1A, right panel). The nuclear and cytoplasmic control proteins, histone and tubulin respectively, were identified in the nuclear and cytoplasmic fractions, respectively, validating the effectiveness of the fractionation (Fig 1A, right panel). Similarly, by fluorescence microscopy, EGFP-P40 localized to the nucleus only in cells infected with AcMNPV expressing polyhedrin (vAc^polh^, diagramed in S1A Fig) (Fig 1B). This phenotype is in accordance with the observation described by Goley *et al*., in which yellow fluorescent protein-tagged P21 (P21-YFP) was observed to accumulate in the nucleus during AcMNPV infection [23].

To test whether EGFP-P40 associates with other Arp2/3 subunits, Arp2-Ha was co-expressed with EGFP or EGFP-P40 in Sf9 cells, respectively. Western blot assay demonstrated that Arp2-Ha (approx. 46 kDa), EGFP (approx. 27 kDa), and EGFP-P40 (approx. 69 kDa) were present in the whole cell lysates (WCL) (Fig 1C, left panel). A co-immunoprecipitation (Co-IP) assay using anti-Ha showed that EGFP-P40, but not EGFP, was pulled down with Arp2-Ha (Fig 1C, left panel), indicating that EGFP-P40 is associated with Arp2-Ha. Similarly, EGFP-P40 is shown to interact with P20-Ha (approx. 21 kDa) (Fig 1C, right panel), implying that EGFP fusion to P40 does not impair the incorporation of P40 into Arp2/3. Taken together, these phenotypes demonstrated that either the C-terminally tagged P40-V_5_ or the N-terminally tagged EGFP-P40 can be used to monitor Arp2/3 dynamics during AcMNPV infection.

We next investigated which class of viral genes needed to be expressed for P40 nuclear accumulation. Aphidicoline (APH), an inhibitor of DNA synthesis, was used to shut off AcMNPV late gene expression [35]. The dynamic localization of P40 in AcMNPV-infected cells was monitored in the presence or absence of APH. Early during infection (0–12 hpi), P40 resided predominantly in the cytoplasm irrespective of APH treatment (Fig 1D). During the late phase of infection (After 12 hpi), AcMNPV infection resulted in detectable P40 accumulation in the nucleus in the absence of APH, suggesting that viral late gene products may play an important role in P40 nuclear accumulation. When the expression of viral late genes was shut off by APH, P40 failed to accumulate in the nucleus, as demonstrated by both immunofluorescence microscopy and cell fractionation assays (Fig 1D). Together, these data indicated that viral late gene products are responsible for P40 nuclear accumulation.

### Ac34 is involved in Arp2/3 nuclear accumulation induced by AcMNPV

**Fig 2:**
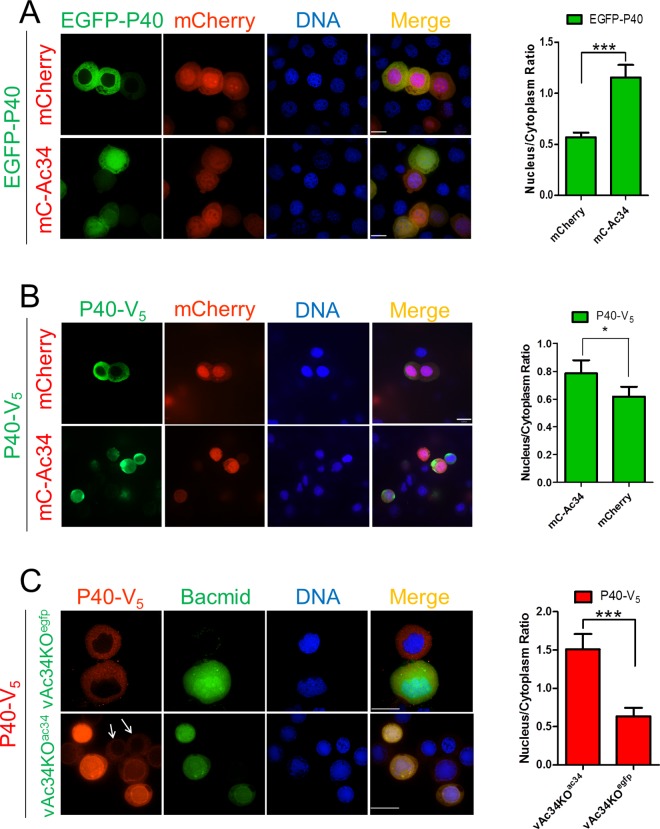
Ac34 induces P40 nuclear accumulation. (A, B). P40 subcellular location in plasmid-transfected cells. EGFP-P40 (A) or P40-V_5_ (B) were transiently co-expressed with mCherry or mCherry-tagged Ac34 (mC-Ac34) in Sf9 cells by plasmid transfection. At 48 hpt, the cells were subjected to fluorescence microscopy assays. (C). P40 subcellular location in bacmid-transfected cells. Sf9 cells expressing P40-V_5_ were co-transfected with vAc34KO^egfp^ or vAc34KO^ac34^. At 48 hpt, the cells were fixed and analyzed by fluorescence microscopy. The arrows pointed to the bacmid-free cells that showed different spatial pattern of P40-V_5_ in comparison with the adjacent cells bearing vAc34KO^ac34^. Densitometry assays were performed simultaneously. The bars represent the means and standard errors of the means for three independent experiments. Each experiment involves the quantification of 30 plasmid- or bacmid-transfected cells. Scale bar: 20 μm. ***, *P<0*.*001*, *, *P<0*.*05*.

To identify the viral protein responsible for the nuclear accumulation of P40, AcMNPV ORFs were individually cloned into a pIZ-V_5_ transient expression vector (Invitrogen). Each individual viral ORF was co-expressed with EGFP-P40 and the subcellular distribution of P40 was determined using fluorescence microscopy. Among the 118 viral ORFs screened (S1 Table), only Ac34, a viral late gene product, appeared to be sufficient to induce P40 nuclear accumulation. Ac34 tagged with mCherry (mC-Ac34) was shown to accumulate EGFP-P40 or P40-V_5_ in the nucleus when co-expressed in Sf9 cells (Fig 2A and 2B). As a control, we co-expressed P40 with non-fused mCherry (Fig 2A and 2B), resulting in a predominantly cytoplasmic localization of P40. Similar nuclear relocation induced by Ac34 also occurred for P20 (S2A Fig), indicating that Ac34 is sufficient to accumulate Arp2/3 in the nucleus.

To further verify the role of Ac34 in P40 nuclear accumulation during AcMNPV infection, an *ac34*-knockout bacmid with an EGFP expression cassette (vAc34KO^egfp^, diagramed in S1B Fig) was constructed [36]. Immunofluorescence microscopy at 48 hours post-transfection (hpt) demonstrated that exogenous P40 (P40-V_5_) resided in the cytoplasm of vAc34KO^egfp^-transfected cells, whereas the restoration of *ac34* to vAc34KO^egfp^ (vAc34KO^ac34^, diagramed in S1B Fig) could accumulate P40-V_5_ in the nucleus (Fig 2C). Similar nuclear accumulation also occurred for P20 (S2B Fig), indicating that *ac34* is responsible for the Arp2/3 nuclear accumulation induced by AcMNPV.

### Arp2/3 nuclear accumulation is dependent on the presence of Ac34 in the nucleus

Previously, we revealed that virus-encoded NPF P78/83, another key element of the nuclear actin polymerization machinery during AcMNPV infection, is relocated to the nucleus by binding to and co-transportation with C42, which harbors a nuclear localization sequence (NLS) [31]. Based on this scenario and the fact that Ac34 is present in the nucleus (Fig 2A and 2B), we were prompted to explore whether P40 nuclear accumulation is also correlated to the presence of Ac34 in the nucleus.

**Fig 3:**
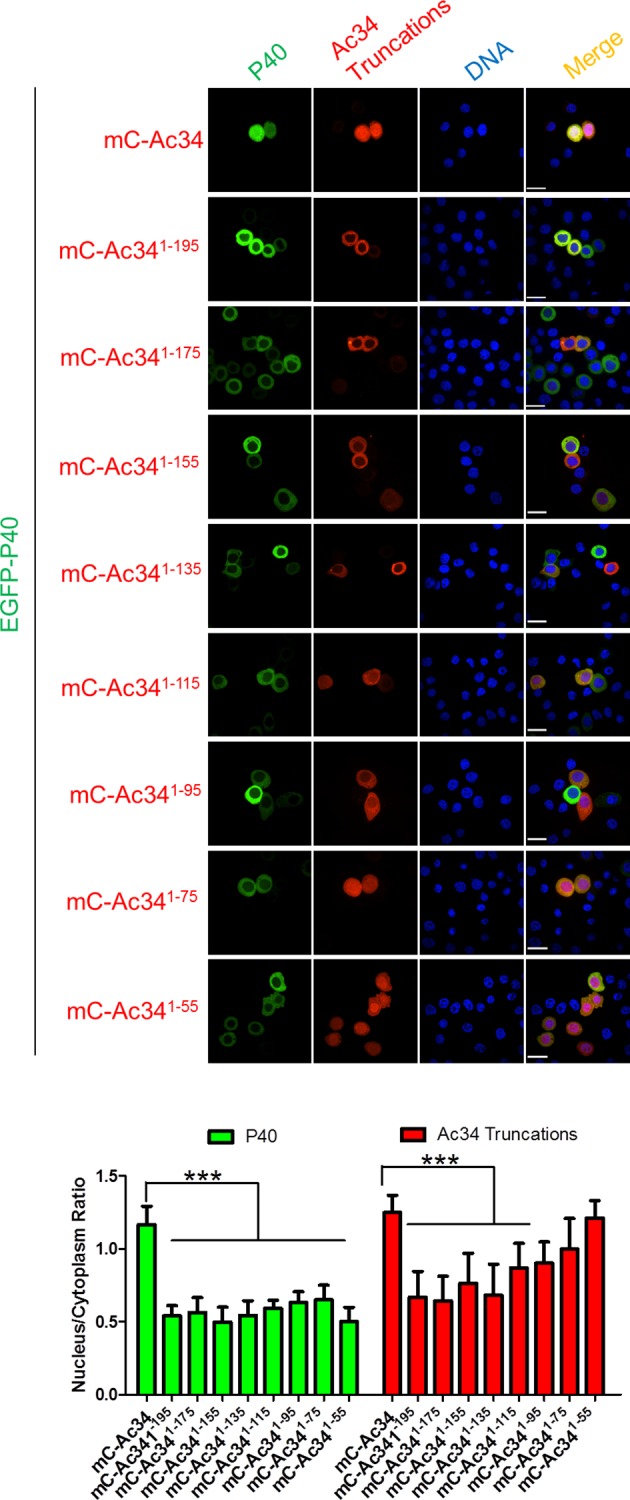
P40 nuclear accumulation is dependent on Ac34 nuclear localization. Subcellular distribution of Ac34 truncations and P40. EGFP-P40 was transiently co-expressed with the mCherry-fused Ac34 truncations in Sf9 cells. At 48 hpt, the cells were analyzed by fluorescence microscopy and densitometry assays were performed simultaneously. The bars represent the means and standard errors of the means for three independent experiments. Each experiment involves the quantification of 30 cells transfected with each plasmid. Scale bar: 20 μm. ***, *P<0*.*001*.

A series of mCherry-fused C-terminal and N-terminal Ac34 truncations (S1C Fig) was prepared to identify the sequence responsible for Ac34 nuclear localization. Fluorescence microscopy demonstrated that the removal of amino acids (aa) 195–215 of Ac34 (mC-Ac34^1-195^) resulted in the cytoplasmic localization of Ac34 (Fig 3), which is in sharp contrast to the full-length Ac34 (mC-Ac34) and all the tested N-terminal Ac34 truncations, which exhibited a predominantly nuclear localization pattern (S3 Fig). This phenotype indicated that the aa 195–215 region plays a major role in determining the presence of Ac34 in the nucleus, although sequence analysis did not show any classic NLS pattern (tandem repeats of lysine and arginine) within this region. Notably, when the C-terminal truncation of Ac34 was extended to aa 75 or further (mC-Ac34^1-75^ and mC-Ac34^1-55^), a diffuse cellular distribution of Ac34 was observed (Fig 3), which could be attributed to free nucleo-cytoplasmic shuttling of the resulting low-molecular-mass polypeptides.

Interestingly, among all the tested Ac34 truncations, only full-length Ac34 could accumulate EGFP-P40 in the nucleus, and the removal of aa 195–215 of Ac34 resulted in a lack of EGFP-P40 nuclear accumulation (Fig 3), thus supporting our hypothesis that P40 nuclear accumulation is dependent on the presence of Ac34 in the nucleus. Similar nuclear accumulation also occurred for P20 (S2A Fig), indicating that the aa 195–215 region is essential for Ac34 to accumulate Arp2/3 in the nucleus.

**Fig 4:**
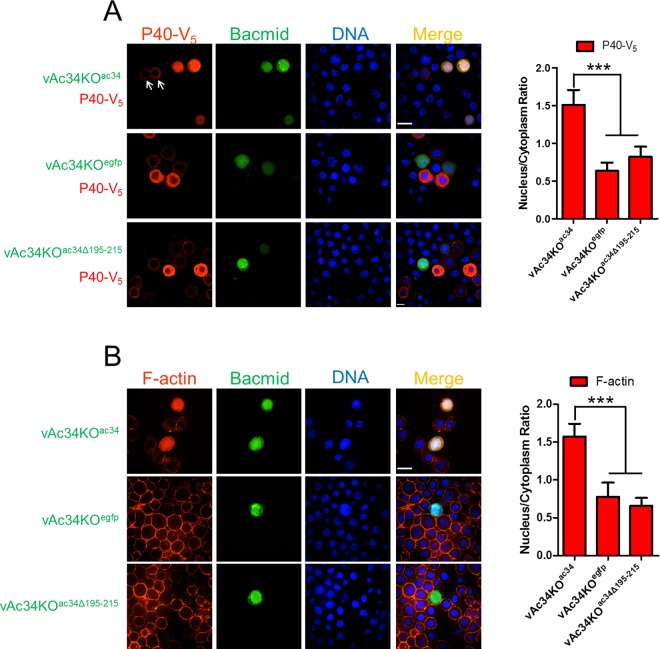
The Ac34 C-terminus is essential for P40 nuclear accumulation and actin polymerization in AcMNPV-infected cells. (A). Ac34 is responsible for P40 nuclear accumulation induced by AcMNPV. Sf9 cells were co-transfected with the indicated bacmids and a P40-V_5_ encoding plasmid and analyzed by immunofluorescence microscopy at 48 hpt. EGFP expression indicated the bacmid-transfected cells, and anti-V_5_ was used to identify the localization of P40. The arrows pointed to the bacmid-free cells that showed different spatial pattern of P40-V_5_ in comparison with the adjacent cells bearing vAc34KO^ac34^. (B). Ac34 is involved in nuclear actin polymerization in virus-infected cells. Sf9 cells were transfected with the indicated bacmids. At 48 hpt, the cells were stained with Alexa Fluor 568-phalloidin and Hoechst and analyzed by fluorescence microscopy. For all the cell images, densitometry assays were performed simultaneously. The bars represent the means and standard errors of the means for three independent experiments. Each experiment involves the quantification of 30 transfected cells for each bacmid. Scale bar: 20 μm. ***, *P<0*.*001*.

To verify the role of aa 195–215 of Ac34 in P40 nuclear accumulation during AcMNPV infection, Ac34^1-195^ was used to rescue vAc34KO^egfp^, generating vAc34KO^ac34Δ195–215^ (diagramed in S1B Fig). When P40 was co-expressed in bacmid-transfected cells, only vAc34KO^ac34^ could induce P40 nuclear accumulation at 48 hpt, in contrast to the cytoplasmic distribution pattern of P40 in vAc34KO^egfp^ and vAc34KO^ac34Δ195-215^-transfected cells (Fig 4A). Similar nuclear accumulation also occurred for P20 (S2B Fig), further confirming that Ac34 is responsible for the Arp2/3 nuclear accumulation induced by AcMNPV, and aa 195–215 are required for the accumulation.

Nuclear actin polymerization requires the nuclear localization of Arp2/3. To explore whether Ac34 is involved in AcMNPV-induced nuclear actin polymerization, Sf9 cells were transfected with vAc34KO^egfp^, vAc34KO^ac34^, or vAc34KO^ac34Δ195–215^ and stained with phalloidin at 48 hpt to visualize F-actin. Among all the transfected bacmids, only vAc34KO^ac34^ induced typical nuclear actin polymerization, with F-actin accumulating in the nuclear region (Fig 4B). The cells transfected with the other bacmids showed no significant F-actin accumulation in the nucleus (Fig 4B). This phenotype can easily be attributed to the absence of Arp2/3 in the nucleus due to either *ac34* knockout (vAc34KO^egfp^) or the loss of its nuclear localization determinant (vAc34KO^ac34Δ195–215^).

### Arp2/3 cytoplasmic distribution is controlled by CRM1-dependent nuclear export

CRM1 is a highly versatile transport receptor that mediates the nuclear export of a large number of proteins. Inhibition of CRM1 results in nuclear retention of NES-bearing protein. Bioinformatics assay (LocNES, http://prodata.swmed.edu/LocNES/) [37] predicted that the P40 C-terminus (aa 360–374), a leucine-rich sequence, is a putative NES. We then explored whether the cytoplasmic distribution of P40 is CRM1-dependent.

**Fig 5:**
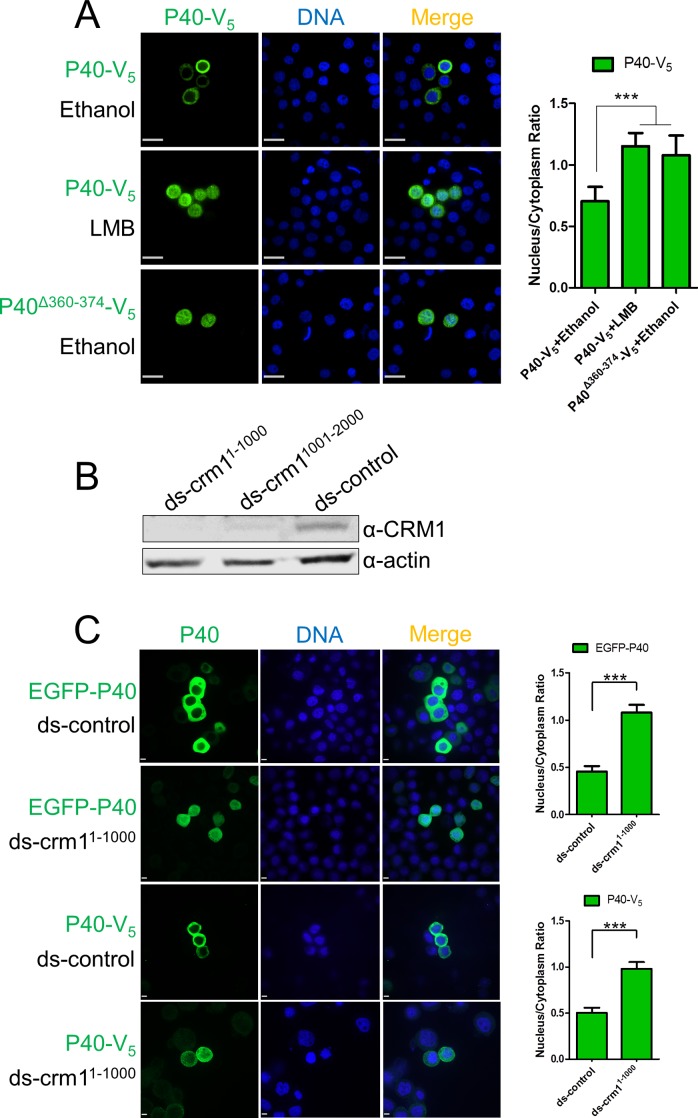
P40 cytoplasmic distribution is controlled by CRM1-dependent nuclear export. (A). P40 cytoplasmic distribution is CRM1-dependent. P40-V_5_ or P40^Δ360-374^-V_5_ was expressed in Sf9 cells. At 44 hpt, the carrier solvent ethanol (1 μl) or an equivalent amount of ethanol containing LMB (0.1 μg/ml) were added to the culture medium. At 48 hpt, the cells were fixed and subjected to immunofluorescence microscopy using anti-V_5_. Scale bar: 20 μm. (B). CRM1 knockdown assay. Sf9 cells were transfected with ds-crm1^1-1000^, ds-crm1^1001-2000^, and ds-control (control dsRNA, included in the MEGAscript RNAi kit). At 72 hpt, cells were harvested and subjected to Western blot assay using anti-CRM1. (C). The impact of CRM1 knockdown on P40 subcellular distribution. Sf9 cells were transfected with ds-crm1^1-1000^ or ds-control. At 24 hpt, plasmids encoding EGFP-P40 and P40-V_5_ were transfected to dsRNA-bearing cells. At 72 hpt, cells were fixed and subjected to fluorescence microscopy assay. Scale bar: 5 μm. Densitometry assays were performed simultaneously. The bars represent the means and standard errors of the means for three independent experiments. Each experiment involves the quantification of 30 transfected cells for each plasmid. ***, *P<0*.*001*.

P40-V_5_ was transiently expressed in Sf9 cells. Immunofluorescence microscopy showed that P40 exhibited significant nuclear accumulation after adding leptomycin B (LMB), a specific CRM1 inhibitor (Fig 5A) [38–40]. Removing aa 360–374 of P40 resulted in P40 (P40^Δ360-374^-V_5_) accumulation in the nucleus (Fig 5A), implying that the P40 C-terminus functions as a NES to determine the cytoplasmic distribution of P40. To further confirm P40 nuclear accumulation is CRM1-dependent, cellular CRM1 was knocked-down using double-stranded RNA (dsRNA) targeting the 1–1000 nt (ds-crm1^1-1000^) or the 1001–2000 nt (ds-crm1^1001-2000^) of CRM1 mRNA (Genbank accession KT208379.1). Western blot assay demonstrated that both dsRNAs significantly down-regulated the endogenous CRM1 level (Fig 5B). Nuclear accumulation of P40-V_5_ and EGFP-P40 was observed in the ds-crm1^1-1000^ bearing cells, in comparison with the control cells (Fig 5C).

Similar nuclear retention upon LMB treatment (S2A Fig) or CRM1 knockdown (S2B Fig) was also observed in P20-expressing cells, indicating that the presence of Arp2/3 in the cytoplasm is controlled by CRM1-dependent nuclear export.

### AcMNPV infection inhibits cellular CRM1-dependent nuclear export

Given the evidence that the cytoplasmic distribution of Arp2/3 is controlled by CRM1-dependent nuclear export, and AcMNPV infection induces Arp2/3 nuclear accumulation, one of the possible explanations is that AcMNPV infection inhibits cellular CRM1-dependent nuclear export and subsequently leads to Arp2/3 retention in the nucleus.

To evaluate the influence of AcMNPV infection on the CRM1 pathway, a classic NES peptide (LQNKLEELDL) [41] was fused to mCherry (mCherry-NES) and EGFP (EGFP-NES) to construct probes for CRM1-dependent nuclear export.

**Fig 6:**
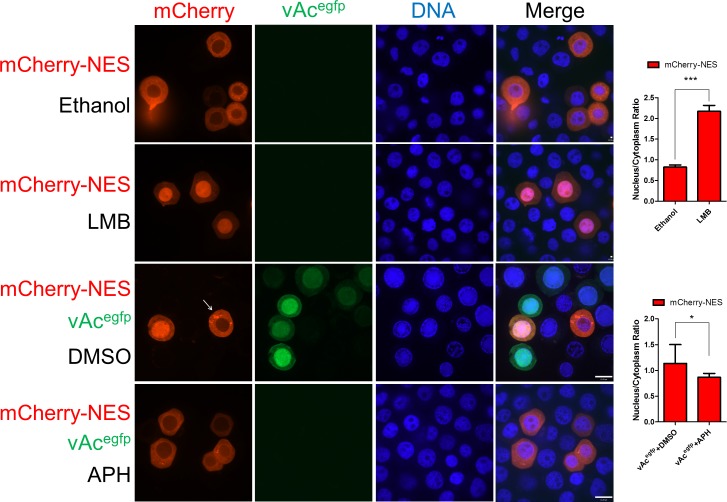
AcMNPV infection inhibits CRM1-dependent nuclear export. mCherry-NES was transiently expressed in Sf9 cells. In virus-free cells (row 1 and 2), ethanol (1 μl) or LMB (0.1 μg/ml) was added to the culture medium at 44 hpt and the cells were incubated for 4 hours; in bacmid-transfected cells (row 3 and 4), the culture medium was discarded and replenished with virus stock solution (vAc^egfp^, MOI = 2) at 24 hpt and incubated for 1 hour. After virus infection, the cells were given fresh medium with a supplement of either DMSO or APH (5 μg/ml). At 48 hpt, all the cells were fixed and subjected to fluorescence microscopy assays. The arrow pointed to the bacmid-free cells that showed different spatial pattern of mCherry-NES in comparison with the adjacent cells bearing vAc^egfp^. Densitometry assays were performed simultaneously. The bars represent the means and standard errors of the means for three independent experiments. Each experiment involves the quantification of 30 transfected cells. Scale bar: 20 μm. ***, *P<0*.*001*, *, *P<0*.*05*.

When mCherry-NES was transiently expressed in Sf9 cells, a predominantly cytoplasmic distribution pattern was observed (Fig 6). Adding LMB to the culture medium resulted in the accumulation of the majority of mCherry-NES in the nucleus (Fig 6), indicating that the nuclear export of mCherry-NES is CRM1-dependent. Similar nuclear retention upon CRM1 knockdown was also observed in EGFP-NES expressing cells (S4 Fig).

When virus stock solution (vAc^egfp^) was added to the culture medium, mCherry-NES accumulated in the nucleus of infected cells and remained in the cytoplasm of uninfected cells (Fig 6). This differential distribution indicated that AcMNPV causes dysfunctional cellular CRM1-dependent nuclear export. To identify which class of viral genes was responsible for the dysfunction, APH was added to the culture medium after virus infection. All the cells showed cytoplasmic distribution of mCherry-NES (Fig 6), suggesting that AcMNPV late gene products were responsible for the virus-induced dysfunction in the CRM1 pathway.

### Ac34 inhibits cellular CRM1-dependent nuclear export during AcMNPV infection

Because we demonstrated that Ac34 is responsible for virus-induced Arp2/3 nuclear accumulation, and AcMNPV inhibits CRM1-dependent nuclear export, which can lead to Arp2/3 retention in the nucleus, it is highly possible that Ac34 is involved in the dysfunction of the CRM1 pathway induced by AcMNPV.

**Fig 7:**
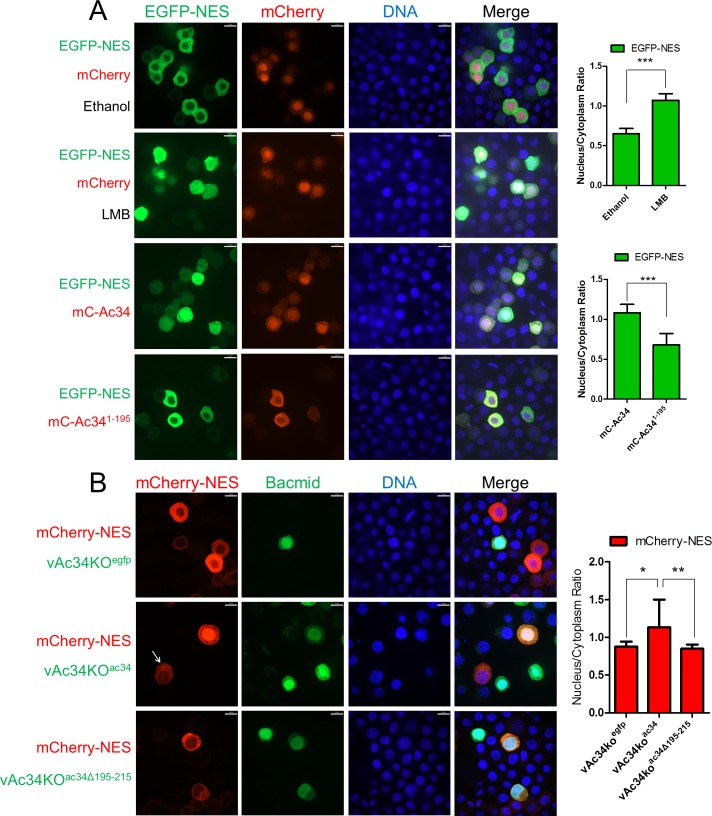
Ac34 inhibits CRM1-dependent nuclear export during AcMNPV infection. (A). Ac34 is sufficient to inhibit CRM1-dependent nuclear export. EGFP-NES was co-expressed with mCherry, mC-Ac34, or mC-Ac34^1-195^ in Sf9 cells. In cells co-expressing EGFP-NES and mCherry (row 1 and 2), ethanol (1 μl) or LMB (0.1 μg/ml) was added to the culture medium at 44 hpt. At 48 hpt, all the cells were fixed and subjected to fluorescence microscopy assays. (B). Ac34 is involved in AcMNPV-induced CRM1 pathway dysfunction. A mCherry-NES encoding plasmid was co-transfected with the indicated bacmids in Sf9 cells. At 48 hpt, all the cells were fixed and subjected to fluorescence microscopy assays. The arrow pointed to the bacmid-free cells that showed different spatial pattern of mCherry-NES in comparison with the adjacent vAc34KO^ac34^ transfected cells. Densitometry assays were performed simultaneously. The bars represent the means and standard errors of the means for three independent experiments. Each experiment involves the quantification of 30 transfected cells. Scale bar: 20 μm. ***, *P<0*.*001*, **, *P<0*.*01* *, *P<0*.*05*.

To test this hypothesis, EGFP-NES was co-expressed with mCherry or mC-Ac34 in Sf9 cells. Fluorescence microscopy showed that EGFP-NES resided in the cytoplasm in the presence of mCherry, whereas it accumulated in the nucleus in the presence of mC-Ac34 or LMB (Fig 7A). This phenotype indicated that Ac34 is sufficient to inhibit CRM1-dependent nuclear export. Removing the Ac34 C-terminus (aa 195–215), which is essential for Ac34 nuclear localization and Arp2/3 nuclear accumulation, also abolished EGFP-NES nuclear retention (Fig 7A).

To validate the role of Ac34 in AcMNPV-induced CRM1 pathway dysfunction, mCherry-NES was co-expressed in bacmid-transfected cells. Fluorescence microscopy showed that mCherry-NES resided in the cytoplasm in vAc34ko^egfp^-transfected cells, whereas the restoration of *ac34* (vAc34ko^ac34^), but not *ac34* ^*Δ195–215*^ (vAc34ko^ac34Δ195–215^), could accumulate mCherry-NES in the nucleus (Fig 7B), indicating that Ac34 is involved in the CRM1 pathway dysfunction induced by AcMNPV.

Taken together, this evidence demonstrated that Ac34 induces Arp2/3 nuclear retention by inhibiting CRM1-dependent nuclear export during AcMNPV infection.

## Discussion

The nuclear import mechanisms of key elements of actin polymerization machinery, including actin and N-WASP, have been previously identified [7–10]. However, nucleo-cytoplasmic shuttling mechanism of Arp2/3, the central regulator of actin polymerization, has not been elucidated yet. In this study, a unique virus-infection system was employed to reveal how Arp2/3 is retained in the nucleus, which could shed light on the nucleo-cytoplasmic shuttling mechanism of Arp2/3 under different physiological or pathophysiological conditions.

Viral manipulation of cellular the nucleo-cytoplasmic transport of proteins has been extensively documented in recent years (reviewed in [42]), in particular in cardioviruses and enteroviruses. Cardioviruses use their leader proteins to induce the hyper-phosphorylation of nucleoporins and disrupt the RanGTP gradient [43, 44], thus inducing an efflux of the nuclear proteins required for viral replication and leading to interferon suppression. Enterovirus infection results in cellular protein retention in the cytoplasm via the degradation of nucleoporins mediated by the virus-encoded proteases 2A and 3C [45–47]. Other viruses, such as herpes simplex virus [48], human papillomavirus [49, 50], severe acute respiratory syndrome coronavirus [51], Ebola virus [52], and measles virus [53], employ a variety of methods to interfere with the nucleo-cytoplasmic shuttling of cellular proteins, therefore facilitating viral replication and escape from the host anti-viral immune response.

Unlike most viruses, which primarily induce impaired protein nuclear import or enhance protein nuclear export, our results demonstrated that AcMNPV infection results in impaired protein nuclear export. As a nucleopolyhedrovirus, most AcMNPV replication processes, including viral genome replication, gene transcription, and nucleocapsid assembly, all occur in the nucleus. These processes require a variety of proteins, including, but not limited to, virus-encoded transcription factors, transcriptases, and capsid proteins, as well as some cellular proteins (e.g., actin, Arp2/3), to accumulate in the nucleus. AcMNPV contains 156 predicted ORFs at least 50 aa in length. Aside from a limited number of exceptions, the nuclear import mechanisms of most viral and cellular proteins during AcMNPV infection remain unknown. Currently, at least 7 exportins have been identified in eukaryotic cells [8, 32, 54–58]. Unlike other exportins that only transport highly specialized cargoes (Reviewer in [59]), CRM1 mediates the nuclear export of many NES-bearing proteins, and its dysfunction leads to the nuclear accumulation of these proteins. Based on bioinformatics prediction (NetNES, http://www.cbs.dtu.dk/services/NetNES/) [60], 98 AcMNPV proteins contain putative residues that could serve as a NES (S2 Table). Such a high percentage of viral proteins bearing putative NESs implies that CRM1-dependent nuclear export may determine the subcellular distribution of many viral proteins, and the inhibition of CRM1-dependent nuclear export by Ac34 could possibly play a key role in the AcMNPV-induced nuclear accumulation of proteins. Whether Ac34 also influences other exportins or these exportins also contribute to the virus-induced protein nuclear accumulation remain to be explored.

Ac34 homologues are presented in all sequenced alphabaculoviruses but absent in betabaculoviruses [61]. Alphabaculoviruses and betabaculoviruses behave in significantly different ways. In respect to cytopathology, alphabaculoviruses assemble their nucleocapsid in the nucleus, whereas betabaculoviruses induce nuclear membrane rupture, and nucleocapsid assembly occurs in a combination of the cytoplasm and the nucleoplasm [62]. This cytopathologic difference suggests that unlike alphabaculoviruses, betabaculoviruses do not need to accumulate the cytoplasmic actin polymerization machinery to the nucleus. As a consequence, betabaculoviruses do not need a viral protein or mechanism to induce nuclear accumulation of Arp2/3 (although only P40 and P20 were proved to be retained in the nucleus of AcMNPV-infected cells in this study, both Arp2/3 components behave in a similar way upon virus infection), which is supported by the evidence that Ac34 homologues are absent in the genomes of betabaculoviruses [61].

Nuclear G-actin is required for the transcriptional activity of RNA polymerases [63–65] and the epigenetic activation of chromatin (Reviewed in [5, 66]). Among the three key actin polymerization elements that are accumulated in the nucleus during AcMNPV infection, only G-actin is recruited to the nucleus by early viral gene products [29, 30]. This early nuclear accumulation of G-actin could increase the nuclear G-actin pool and promote the transcription of viral early genes that are transcribed by host RNA polymerase II [61]. Late in infection, P78/83 and Arp2/3 accumulate in the nucleus and induce nuclear actin polymerization that converts G-actin to F-actin. The resulting nuclear G-actin pool depletion could lead to the loss of the transcriptional activity of host RNA polymerases and the epigenetic reprogramming of host chromatin towards transcriptional inhibition, which could contribute to the host gene transcription shutoff that occurs in the late phase of baculovirus infection [67, 68]. Consistent with this, cytochalasin D, a chemical that specifically prevents actin polymerization, behaves as an antagonist of the virus-induced shutdown of host gene expression [69]. In this respect, nuclear actin polymerization induced by baculovirus infection may also participate in the regulation of host/virus gene expression by the modulation of the nuclear G-actin pool, in addition to its role in assisting viral nucleocapsid assembly and transport, which has long been recognized.

In summary, Ac34 subversion of the CRM1-dependent nuclear export during AcMNPV infection suggests that alphabaculoviruses may employ an efficient way by encoding a single protein to accumulate multiple viral and host proteins in the nucleus to assist in virus replication. As a key element of actin polymerization machinery, Arp2/3 is present in both the cytoplasm and the nucleus. Our finding that Arp2/3 nuclear-cytoplasmic shuttling is CRM1-dependent sheds light on how cells manage to control actin polymerization machinery in different cellular compartments to exert different functions.

## Materials and Methods

### Cell culture, transfection, and infection

Sf9 cells from *S*. *frugiperda* were cultured in Grace’s medium (Invitrogen) with 5% fetal bovine serum (Invitrogen) and 0.1% Antibiotic-Antimycotic (Invitrogen) at 27°C. Sf9 cells were transfected with the indicated plasmids or bacmids using the Cellfectin II reagent (Invitrogen) following the standard procedures. For infection, the Sf9 cells were incubated with virus stock solution for 1 h at a multiplicity of infection (MOI) of 2. (MOI = 2). The cells were then rinsed twice and then incubated in fresh medium or medium with APH (5 μg/ml) (Sigma). The cells were fixed for further immunofluorescence detection at 6, 12, and 24 hpi. To block CRM1-dependent nuclear export, LMB (0.1 μg/ml) (Beyotime) was added to the culture medium and the cells were incubated for 4 hours before the fluorescence assays.

### Construction of AcMNPV ORF transient expression library

One hundred fifty-four ORFs of AcMNPV were cloned by polymerase chain reaction (PCR) and inserted into pIZ-V_5_ (Invitrogen). All the viral ORFs began with ATG and ended without the stop codon to create an in-frame fusion with the V_5_ epitope. All the constructs were sequenced, and 118 viral ORFs were tested for their impact on the change in P40 subcellular distribution (S1 Table).

### Construction of plasmids and bacmids

All the plasmids used in this research for transient expression were prepared by standard molecular cloning protocols. The indicated genes, gene truncations, and genes with epitope tags were generated by PCR or site-directed mutagenesis (Transgene) and inserted into pIZ-V_5_/Ha vectors (Invitrogen).

To prepare recombinant bacmids, the Bac-to-Bac system was employed according to Invitrogen’s protocol. In brief, Ac34 expression cassettes controlled by the native *ac34* promoter were cloned into pFbdg, a pFastbac-Dual vector (Invitrogen) bearing an EGFP expression cassette controlled by the *p10* promoter [31]. The resulting shuttle vectors were then used to transform DH10B *E*. *coli* cells harboring the vAc34KO bacmid provided by Cai *et al*. to generate the transposed bacmid constructs [36].

Maps of the plasmids and bacmids prepared in this research are diagramed in S1 Fig.

### Cell fractionation

Cells were rinsed with ice-cold PBS and lysed with homogenization buffer (10 mM HEPES pH = 7.9, 10 mM KCl, 1.5 mM MgCl_2,_ 0.1 mM EGTA, 0.5 mM DTT, 2 mM PMSF, 1 μg/ml Proteinase Inhibitors (Roche)). The cell membranes were disrupted by passing through a 25G needle 5 times, and the lysates were then spun at 1000×g for 10 min at 4°C. The supernatant containing the crude cytoplasmic fraction was collected in 1.5 ml tubes and spun at 20,817×g for 30 min at 4°C, and the supernatant was collected as the purified cytoplasmic fraction. The nuclear pellet was rinsed in 1 ml homogenization buffer and centrifuged at 1000×g for 10 min at 4°C. The pellet was re-suspended in 100 μl extraction buffer (10 mM HEPES pH = 7.9, 0.4 M NaCl, 1.5 mM MgCl_2_, 0.1 mM EGTA, 0.5 mM DTT, 2 mM PMSF, 1 μg/ml Proteinase Inhibitors) under gentle shaking for 30 min at 4°C. The suspension was centrifuged at 20,817×g for 30 min at 4°C and the supernatant was collected as the nuclear fraction. The protein concentrations of all samples were determined using Bradford assays (Bio-Rad) and the samples were subjected to Western blot assays. Anti-histone H3 (Sigma) and anti-tubulin (Sigma) diluted to 1:1000 were used to verify the quality of the cytoplasmic and nuclear fractions, respectively. After HRP-conjugated secondary antibody (1:10,000 dilution, Jackson Laboratory) incubation, the blots were developed using an enhanced chemiluminescence kit (Pierce).

### Immunoprecipitation

Sf9 cells were rinsed with ice-cold PBS and lysed with RIPA buffer (50 mM Tris, pH = 7.5, 1 mM EGTA, 1 mM EDTA, 1% Triton X-100, 150 mM NaCl, 2 mM DTT, 100 μM PMSF, 1 μg/ml Proteinase Inhibitors). The cell lysates were centrifuged at 20,817×g at 4°C for 10 min and the supernatants (WCL) were collected. The protein concentrations of the WCL were determined by Bradford assays and 1500 μg was mixed with 2 μg anti-Ha (Sigma) and Protein G Agarose (Millipore) and incubated at 4°C overnight according to the manufacturer’s protocol. The immunoprecipitated samples were centrifuged and washed three times and subjected to Western blot assays using anti-Ha (1:1000 dilution) and anti-EGFP (1:1000 dilution, Invitrogen).

### Immunofluorescence assay and F-actin staining

The immunofluorescence assays were performed as described previously [31]. Briefly, the cells were fixed with 3.7% paraformaldehyde in PBS for 30 min, permeabilized with 0.5% Triton X-100 and blocked in 1% normal goat serum (Boster) in PBS for 30 min on ice. The cells were incubated with anti-V_5_ (1:500 dilution, Invitrogen) or anti-Ha (1:500 dilution, Sigma) primary antibodies. The secondary antibodies were Alexa Fluor 568- or 488-conjugated anti-mouse and anti-rabbit antibodies (1:500 dilution, Invitrogen). The nuclear DNA was stained with Hoechst 33258 (Beyotime).

For F-actin staining, the cells were transfected with different recombinant bacmids, fixed, and permeabilized as described above and then stained with 0.7 U/ml Alexa Fluor 568-phalloidin (Invitrogen) and Hoechst 33258 for 10 min. The cells were then washed three times with PBS and examined by confocal microscopy using a PerkinElmer UltraVIEW VoX microscope.

### Fluorescence quantification and statistical assays

The fluorescence quantification data were obtained using Volocity 6.3 software (PerkinElmer) and Student’s T-test was performed to compare the differences between the tested samples.

### CRM1 knockdown assay

To knockdown the expression of CRM1, primers encompassing the 1–1000 nt (TAATACGACTCACTATAGGGATGGCAACTTTAGAGCAACA, TAATACGACTCACTATAGGGACTTCAGATATCAGTACAAG) or the 1001–2000 nt (TAATACGACTCACTATAGGGAGAAGAAGTAGAAATTTTTA, TAATACGACTCACTATAGGGTGTCCAAATATATTCTACCC) of *S*. *frugiperda* CRM1 mRNA (Genbank accession: KT208379.1) were synthesized and served as gene specific primers to prepare dsRNA by using MEGAscript RNAi kit (Ambion) according to the manufacturer’s protocols. Sf9 cells were transfected with 5 μg dsRNA/10^5^ cells using the Cellfectin II reagent (Invitrogen).

## Supporting Information

S1 Fig Diagrams of plasmids and bacmids. (A). bMON14272-based bacmids. The coding sequences of EGFP or Polyhedrin were inserted into the downstream region of the *p10* promoter (*P*_*p10*_) or the *polyhedrin* promoter (*Ppolh*). The resulting plasmids were transposed into bMON14272 (Invitrogen), a bacmid harboring the wild-type AcMNPV genome, at the *polyhedrin* (*polh*) locus using the Bac-to-Bac method. (B). vAc34KO-based bacmids. The coding sequences of EGFP, Ac34, and Ac34^1-195^ were inserted into the downstream region of the *p10* promoter (*P*_*p10*_) or the *ac34* promoter (*P*_*ac34*_). The resulting plasmids were transposed into vAc34KO at the *polyhedrin* (*polh*) locus using the Bac-to-Bac method. (C). N-terminal or C-terminal Ac34 truncations were fused to mCherry. The expression of the fusion proteins was controlled by an *OpIE2* promoter (*P*_*opIE2*_).
(TIF)

S2 Fig Ac34 mediates P20 nuclear relocation. (A). Ac34 is sufficient to relocate P20 to the nucleus. P20-Ha was co-expressed with mCherry, mC-Ac34, or mC-Ac34^1-195^ in Sf9 cells. At 44 hpt, LMB (0.1 μg/ml) was added to the culture medium and the cells were incubated for 4 hours. At 48 hpt, all the cells were fixed and subjected to immunofluorescence microscopy assays using anti-Ha. Scale bar: 20 μm. (B). The impact of CRM1 knockdown on P20 subcellular distribution. Sf9 cells were transfected with ds-crm1^1-1000^ or ds-control. At 24 hpt, plasmids encoding P20-Ha were transfected to dsRNA-bearing cells. At 72 hpt, cells were fixed and subjected to immunofluorescence microscopy assay using anti-Ha. Scale bar: 5 μm. (C). Ac34 is involved in P20 nuclear relocation induced by AcMNPV. Plasmids encoding P20-Ha were co-transfected with vAc^egfp^, vAc34KO^egfp^, or vAc34KO^ac34Δ195–215^. At 48 hpt, the cells were fixed and subjected to immunofluorescence microscopy assays using anti-Ha. The arrow pointed to the bacmid-free cells that showed different spatial pattern of P20-Ha in comparison with the adjacent cells bearing vAc34KO^ac34^. Scale bar: 20 μm. Densitometry assays were performed simultaneously. The bars represent the means and standard errors of the means for three independent experiments. Each experiment involves the quantification of 30 transfected cells. ***, *P<0*.*001*.
(TIF)

S3 Fig Subcellular distribution of Ac34 N-terminal truncations. A series of mCherry-fused Ac34 N-terminal truncations were transiently expressed in Sf9 cells. At 48 hpt, the cells were fixed and subjected to fluorescence microscopy assays. Densitometry assays were performed simultaneously. The bars represent the means and standard errors of the means for three independent experiments. Each experiment involves the quantification of 30 transfected cells. Scale bar: 20 μm.
(TIF)

S4 Fig The impact of CRM1 knockdown on CRM1-dependent nuclear export. Sf9 cells were transfected with ds-control and ds-crm1^1-1000^. At 24 hpt, plasmids encoding EGFP-NES were transfected to the dsRNA-bearing cells. At 72 hpt, cells were fixed and subjected to florescence microscopy assay. Densitometry assays were performed simultaneously. The bars represent the means and standard errors of the means for three independent experiments. Each experiment involves the quantification of 30 transfected cells. Scale bar: 5 μm.
(TIF)

S1 Table Tested and un-tested viral ORFs of the AcMNPV library. A total of 154 ORFs of AcMNPV were cloned by PCR and inserted into pIZ-V_5_ to generate a transient expression library. A total of 118 viral ORFs were tested to evaluate their impact on EGFP-P40 subcellular distribution.
(DOCX)

S2 Table Predicted NES-containing proteins encoded by AcMNPV. Amino acid sequences of AcMNPV (Genbank accession NC_001623.1) encoded proteins were submitted to NetNES (http://www.cbs.dtu.dk/services/NetNES/) to predict leucine-rich NES.
(XLSX)
