# Supplementary figures and images for: Autographa californica Multiple Nucleopolyhedrovirus Ac34 Protein Retains Cellular Actin-Related Protein 2/3 Complex in the Nucleus by Subversion of CRM1-Dependent Nuclear Export
Source: PLoS Pathog. 2016 Nov 1;12(11):e1005994. doi: 10.1371/journal.ppat.1005994 (PMC5089780; doi:10.1371/journal.ppat.1005994)

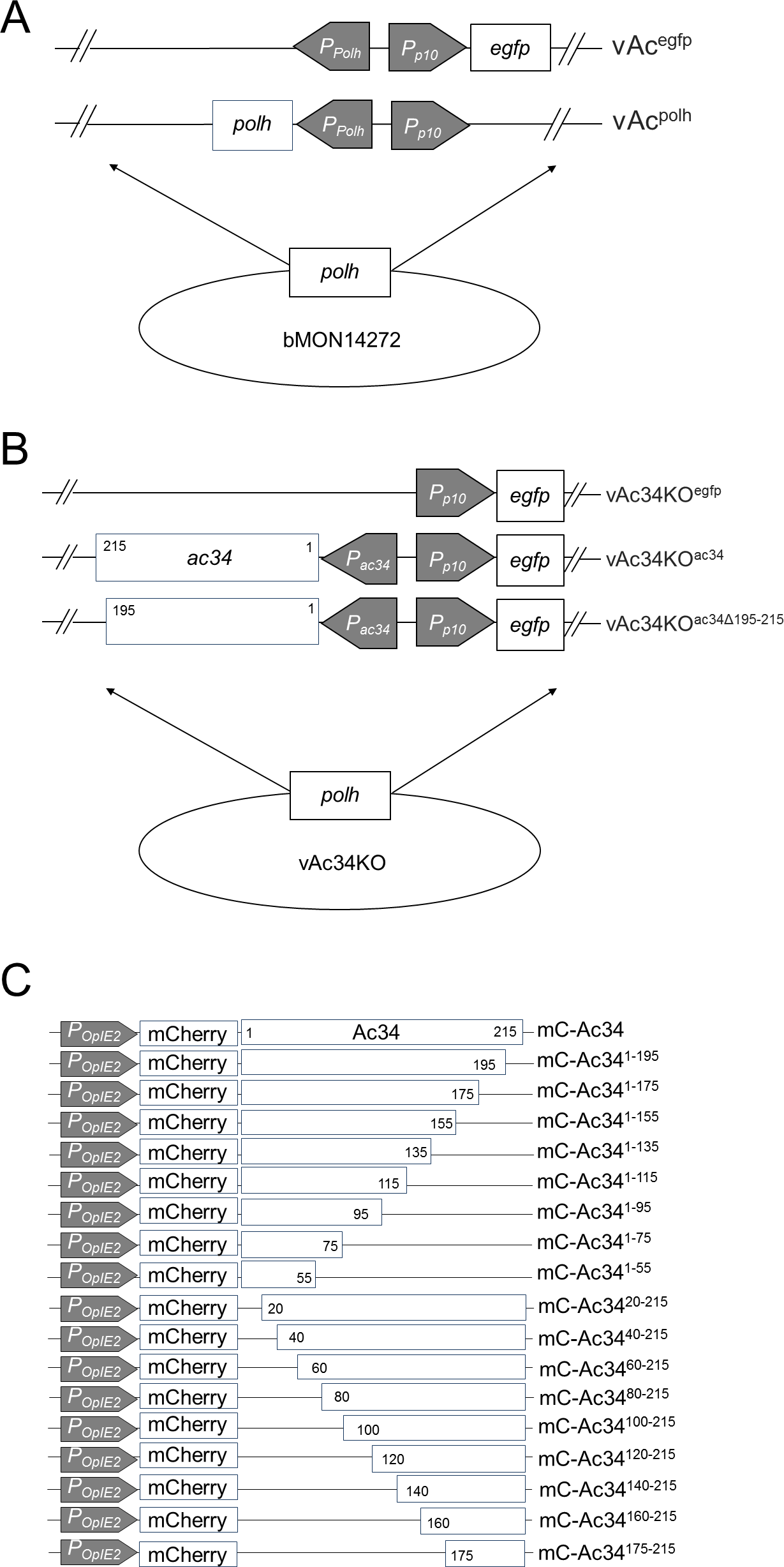

Supplement: S1 Fig — (A). bMON14272-based bacmids. The coding sequences of EGFP or Polyhedrin were inserted into the downstream region of the p10 promoter (P p10) or the polyhedrin promoter (Ppolh). The resulting plasmids were transposed into bMON14272 (Invitrogen), a bacmid harboring the wild-type AcMNPV genome, at the polyhedrin (polh) locus using the Bac-to-Bac method. (B). vAc34KO-based bacmids. The coding sequences of EGFP, Ac34, and Ac341-195 were inserted into the downstream region of the p10 promoter (P p10) or the ac34 promoter (P ac34). The resulting plasmids were transposed into vAc34KO at the polyhedrin (polh) locus using the Bac-to-Bac method. (C). N-terminal or C-terminal Ac34 truncations were fused to mCherry. The expression of the fusion proteins was controlled by an OpIE2 promoter (P opIE2). (TIF) [file ppat.1005994.s001.tif]

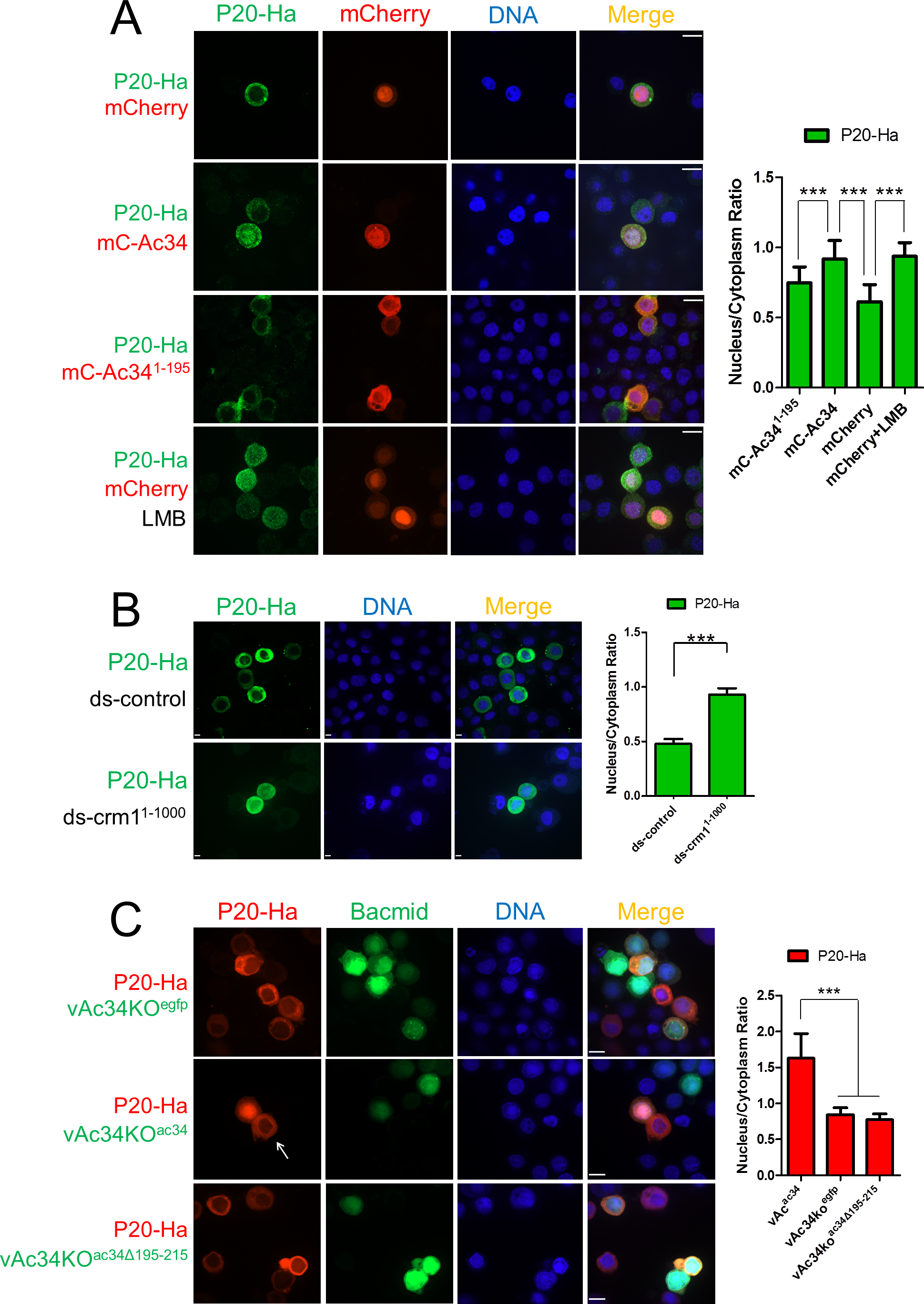

Supplement: S2 Fig — (A). Ac34 is sufficient to relocate P20 to the nucleus. P20-Ha was co-expressed with mCherry, mC-Ac34, or mC-Ac341-195 in Sf9 cells. At 44 hpt, LMB (0.1 μg/ml) was added to the culture medium and the cells were incubated for 4 hours. At 48 hpt, all the cells were fixed and subjected to immunofluorescence microscopy assays using anti-Ha. Scale bar: 20 μm. (B). The impact of CRM1 knockdown on P20 subcellular distribution. Sf9 cells were transfected with ds-crm11-1000 or ds-control. At 24 hpt, plasmids encoding P20-Ha were transfected to dsRNA-bearing cells. At 72 hpt, cells were fixed and subjected to immunofluorescence microscopy assay using anti-Ha. Scale bar: 5 μm. (C). Ac34 is involved in P20 nuclear relocation induced by AcMNPV. Plasmids encoding P20-Ha were co-transfected with vAcegfp, vAc34KOegfp, or vAc34KOac34Δ195–215. At 48 hpt, the cells were fixed and subjected to immunofluorescence microscopy assays using anti-Ha. The arrow pointed to the bacmid-free cells that showed different spatial pattern of P20-Ha in comparison with the adjacent cells bearing vAc34KOac34. Scale bar: 20 μm. Densitometry assays were performed simultaneously. The bars represent the means and standard errors of the means for three independent experiments. Each experiment involves the quantification of 30 transfected cells. ***, P<0.001. (TIF) [file ppat.1005994.s002.tif]

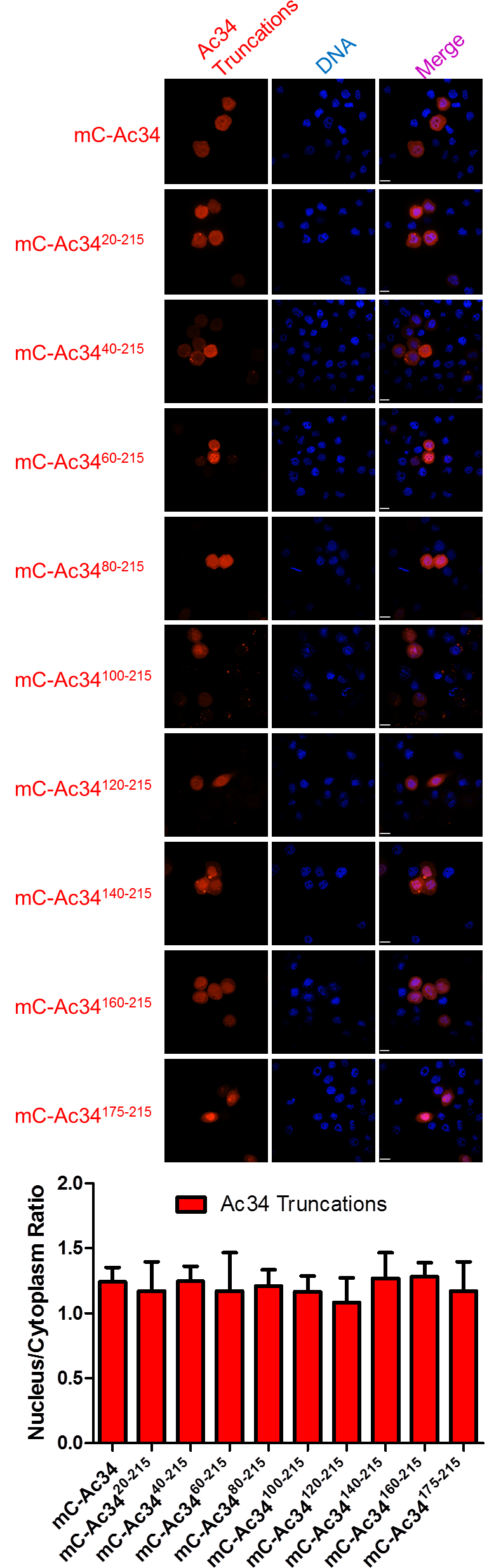

Supplement: S3 Fig — A series of mCherry-fused Ac34 N-terminal truncations were transiently expressed in Sf9 cells. At 48 hpt, the cells were fixed and subjected to fluorescence microscopy assays. Densitometry assays were performed simultaneously. The bars represent the means and standard errors of the means for three independent experiments. Each experiment involves the quantification of 30 transfected cells. Scale bar: 20 μm. (TIF) [file ppat.1005994.s003.tif]

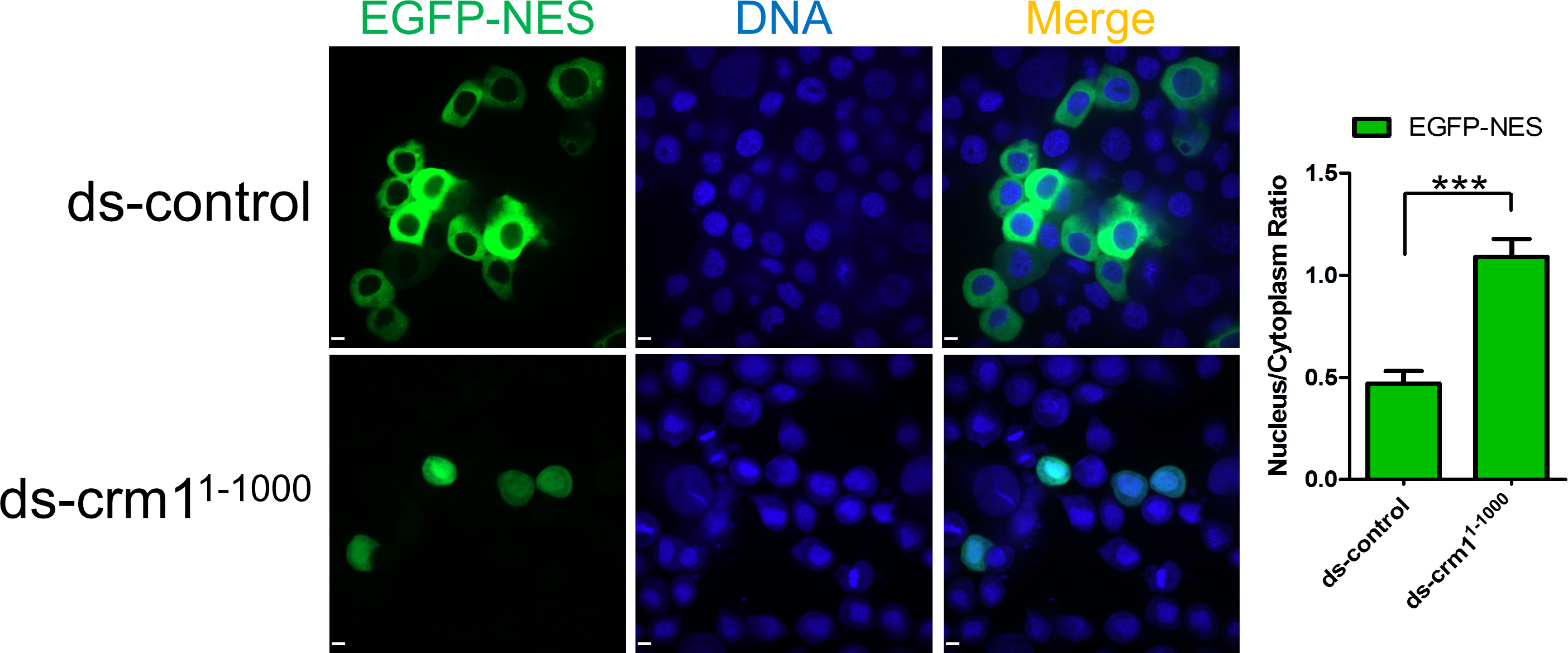

Supplement: S4 Fig — Sf9 cells were transfected with ds-control and ds-crm11-1000. At 24 hpt, plasmids encoding EGFP-NES were transfected to the dsRNA-bearing cells. At 72 hpt, cells were fixed and subjected to florescence microscopy assay. Densitometry assays were performed simultaneously. The bars represent the means and standard errors of the means for three independent experiments. Each experiment involves the quantification of 30 transfected cells. Scale bar: 5 μm. (TIF) [file ppat.1005994.s004.tif]
