# Supplementary material for: Autographa californica Multiple Nucleopolyhedrovirus Ac34 Protein Retains Cellular Actin-Related Protein 2/3 Complex in the Nucleus by Subversion of CRM1-Dependent Nuclear Export
Source: PLoS Pathog. 2016 Nov 1;12(11):e1005994. doi: 10.1371/journal.ppat.1005994 (PMC5089780; doi:10.1371/journal.ppat.1005994)
Supplement: S1 Table — A total of 154 ORFs of AcMNPV were cloned by PCR and inserted into pIZ-V5 to generate a transient expression library. A total of 118 viral ORFs were tested to evaluate their impact on EGFP-P40 subcellular distribution. (DOCX) [file ppat.1005994.s005.docx]

| Tested ORFs | | | | Un-tested ORFs |
| --- | --- | --- | --- | --- |
| AcOrf-1 | AcOrf-45 | AcOrf-93 | AcOrf-137 | AcOrf-10 |
| AcOrf-2 | AcOrf-47 | AcOrf-94 | AcOrf-138 | AcOrf-15 |
| AcOrf-3 | AcOrf-49 | AcOrf-96 | AcOrf-139 | AcOrf-19 |
| AcOrf-4 | AcOrf-50 | AcOrf-97 | AcOrf-142 | AcOrf-20 |
| AcOrf-5 | AcOrf-51 | AcOrf-98 | AcOrf-143 | AcOrf-24 |
| AcOrf-6 | AcOrf-52 | AcOrf-99 | AcOrf-144 | AcOrf-26 |
| AcOrf-7 | AcOrf-53 | AcOrf-100 | AcOrf-149 | AcOrf-27 |
| AcOrf-8 | AcOrf-54 | AcOrf-101 | AcOrf-150 | AcOrf-44 |
| AcOrf-9 | AcOrf-55 | AcOrf-102 | AcOrf-153 | AcOrf-46 |
| AcOrf-11 | AcOrf-56 | AcOrf-103 | AcOrf-154 | AcOrf-48 |
| AcOrf-12 | AcOrf-57 | AcOrf-105 |  | AcOrf-61 |
| AcOrf-13 | AcOrf-58 | AcOrf-106 |  | AcOrf-62 |
| AcOrf-14 | AcOrf-59 | AcOrf-107 |  | AcOrf-67 |
| AcOrf-16 | AcOrf-60 | AcOrf-108 |  | AcOrf-72 |
| AcOrf-17 | AcOrf-63 | AcOrf-109 |  | AcOrf-74 |
| AcOrf-18 | AcOrf-64 | AcOrf-110 |  | AcOrf-76 |
| AcOrf-21 | AcOrf-65 | AcOrf-111 |  | AcOrf-85 |
| AcOrf-22 | AcOrf-66 | AcOrf-112 |  | AcOrf-89 |
| AcOrf-23 | AcOrf-68 | AcOrf-113 |  | AcOrf-91 |
| AcOrf-25 | AcOrf-69 | AcOrf-114 |  | AcOrf-92 |
| AcOrf-28 | AcOrf-70 | AcOrf-116 |  | AcOrf-95 |
| AcOrf-29 | AcOrf-71 | AcOrf-118 |  | AcOrf-104 |
| AcOrf-30 | AcOrf-73 | AcOrf-119 |  | AcOrf-115 |
| AcOrf-31 | AcOrf-75 | AcOrf-120 |  | AcOrf-117 |
| AcOrf-32 | AcOrf-77 | AcOrf-121 |  | AcOrf-126 |
| AcOrf-33 | AcOrf-78 | AcOrf-122 |  | AcOrf-133 |
| AcOrf-34 | AcOrf-79 | AcOrf-123 |  | AcOrf-134 |
| AcOrf-35 | AcOrf-80 | AcOrf-124 |  | AcOrf-135 |
| AcOrf-36 | AcOrf-81 | AcOrf-125 |  | AcOrf-140 |
| AcOrf-37 | AcOrf-82 | AcOrf-127 |  | AcOrf-141 |
| AcOrf-38 | AcOrf-83 | AcOrf-128 |  | AcOrf-145 |
| AcOrf-39 | AcOrf-84 | AcOrf-129 |  | AcOrf-146 |
| AcOrf-40 | AcOrf-86 | AcOrf-130 |  | AcOrf-147 |
| AcOrf-41 | AcOrf-87 | AcOrf-131 |  | AcOrf-148 |
| AcOrf-42 | AcOrf-88 | AcOrf-132 |  | AcOrf-151 |
| AcOrf-43 | AcOrf-90 | AcOrf-136 |  | AcOrf-152 |
